# Maternal Supplementation with Dietary Betaine during Gestation to Improve Twin Lamb Survival

**DOI:** 10.3390/ani10101749

**Published:** 2020-09-26

**Authors:** Billie-Jaye Brougham, Alice C. Weaver, Alyce M.F. Swinbourne, Bobbie E. Lewis Baida, Jennifer M. Kelly, Simon K. Walker, David O. Kleemann, William H.E.J. van Wettere

**Affiliations:** 1 Davies Livestock Research Centre, School of Animal and Veterinary Sciences, The University of Adelaide, Roseworthy, SA 5371, Australia; alyce.swinbourne@adelaide.edu.au (A.M.S.); bobbie.lewisbaida@adelaide.edu.au (B.E.L.B.); william.vanwettere@adelaide.edu.au (W.H.v.W.); 2 South Australian Research and Development Institute, Turretfield Research Centre, Rosedale, SA 5350, Australia; Alice.Weaver@sa.gov.au (A.C.W.); Jen.Kelly@sa.gov.au (J.M.K.); walkersa@adam.com.au (S.K.W.); Dave.Kleemann@sa.gov.au (D.O.K.)

**Keywords:** betaine, sheep, maternal supplement, neonatal mortality, lamb survival

## Abstract

**Simple Summary:**

High incidences of twin lamb mortality constrain the reproductive efficiency and productivity of Merino sheep flocks. This study determined whether supplementing the diets of pregnant, twin-bearing ewes with 2 or 4 g/day of betaine would improve lamb viability and survival to weaning. Feeding ewes 2 g/day of betaine for the duration of pregnancy decreased lamb survival but increased lamb body weight at weaning. Whereas, lamb vigour and early post-natal survival were improved following ewe supplementation with 4 g/day of betaine during the second half of pregnancy. Maternal supplementation with 4 g/day of betaine during the second half of pregnancy may, therefore, be a useful strategy to improve twin lamb survival.

**Abstract:**

Betaine increases the synthesis of creatine, an energy-rich amino acid that increases adenosine triphosphate (ATP) and has neuroprotective properties which may improve post-natal lamb survival. This study determined whether maternal betaine supplementation during gestation would improve body weight, thermoregulation, time to stand and suck, colostrum intake and survival to weaning of twin lambs. Twin-bearing Merino ewes received dietary betaine at either 0 g/day (Control, CTL), 2 g/day from ram introduction to parturition (Early betaine, EB) or 4 g/day from Day 80 of gestation to parturition (Late betaine, LB). Ewes were housed individually during parturition and measures were collected at 4, 24 and 72 h and Day 7 post-partum, and at marking (53.2 ± 0.2 days of age) and weaning (99.3 ± 0.2 days of age). The EB treatment resulted in heavier lambs at weaning compared with CTL and LB lambs (*p* < 0.05). Time to stand and suck from birth was longer in EB lambs (*p* < 0.05), whereas, the interval from birth to first suck was shorter for LB lambs (*p* < 0.05). Lamb survival rate was the highest for LB lambs at 72 h and Day 7 (*p* < 0.05), and lowest for EB lambs on Day 7 (*p* < 0.05). These data indicated that betaine supplementation at 4 g/day during the second half of pregnancy improved twin lamb survival to Day 7 and shortened the interval from birth to first suck; whereas feeding ewes 2 g/day of betaine for the duration of pregnancy increased twin lamb body weight at weaning, but increased both the time to attain behavioural milestones and mortalities before Day 7.

## 1. Introduction

Approximately 17 million lambs die annually in Australia [1] with twin lambs representing 35.3% of total reproductive loss between ovulation and weaning [2]. This equates to an estimated loss of around $540 million per annum [3] and represents a significant economic and welfare concern. Lambs are most vulnerable during the first 72 h of their life, with 43 to 70% of perinatal deaths occurring during this period [4,5,6]. Approximately 50% of deaths arise from complications occurring in utero and/or immediately following birth: stillbirth, dystocia and hypoxic birth injury [5,6]. Twin-born lambs are lighter at birth [7] and often have impaired thermoregulatory ability due to poor accumulation of energy reserves [8]. Together these factors decrease lamb vigour and consequently delay the standing and sucking responses, making them more likely to die from starvation and/or hypothermia [9,10].

Supplementing pregnant ewes with betaine has the potential to improve neonatal twin lamb survival. Betaine is a trimethyl derivative of the amino acid glycine and diffuses across the ovine placenta [11,12,13]. Betaine increases the production of creatine via the donation of methyl groups in a transmethylation reaction [11,14,15,16]. Creatine is an energy-rich amino acid that facilitates the recycling of adenosine triphosphate (ATP); the main energy carrier molecule involved in tissue development and growth, particularly in the brain and muscle. Therefore, creatine acts as an endogenous neuroprotectant, protecting the neonatal brain from oxygen deprivation experienced during parturition [17]. In sows, dietary betaine supplemented throughout gestation reduced the levels of plasma homocysteine [18]; an amino acid that has shown to adversely affect embryonic development and increase prenatal mortality in several species [19,20]. Further, supplementary betaine during late gestation increased the number of piglets born alive [18], as well as piglet energy synthesis and glycogen stores at birth [21]. Supplementing creatine to pregnant spiny mice reduced the incidence of neonatal hypoxia due to higher ATP levels resulting from increased creatine supply and its neuroprotective capacity [22,23,24].

Based on the literature described, we suggest that maternal betaine supplementation will increase creatine synthesis by the ovine fetus, and increase energy stores available to the newborn lamb. As a result, incidences of intrapartum deaths (stillbirths) and the adverse effects of hypoxic birth injury will be reduced, resulting in improved lamb vigour, thermoregulation, colostrum intake and, ultimately, survival. To the best of our knowledge, studies directly addressing the effects of maternal supplementation with betaine as a means of improving twin lamb survival have not been explored. Therefore, this experiment examined the effects of maternal supplementation with two different doses (2 or 4 g/ewe/day) and administration times (early or late) of dietary betaine during gestation on twin lamb weight, thermoregulatory capacity, time to stand and suck, colostrum intake and survival from birth to weaning.

## 2. Materials and Methods

This experiment was conducted collaboratively between the University of Adelaide and South Australian Research and Development Institute (SARDI) at the Turretfield Research Centre, Rosedale, South Australia (34°33′ S). All animal procedures were conducted in accordance with the guidelines of the Australian Code of Practice for the Use of Animals for Scientific Purposes and received approval from the Primary Industries and Regions South Australia Animal Ethics Committee (#16/18).

### 2.1. Animal Housing, Management and Feeding Regimes

A total of 240 mature (6 to 9 years), multiparous South Australian Merino ewes (*Ovis aries*) were selected based on weight and condition score (CS; 1 to 5 scale, with 1 = emaciated and 5 = obese, adapted from [25]) and were then randomly allocated to one of three treatment groups to ensure even weight and CS across treatments. Following treatment allocation, ewes were synchronized for oestrus by the insertion of progesterone controlled internal drug release (CIDR) devices (EAZI-BREED™ CIDR^®^ Sheep Insert, 0.3 g progesterone in silicone elastomer, Pharmacia and Upjohn, Rydalmere, New South Wales, Australia). Twelve days later CIDRs were removed and Merino rams, fitted with harness and crayons, were put with the ewes for nine days. Daily crayon marks were recorded to identify day of mating. At approximately Day 55 post-mating, ewes were pregnancy scanned by transabdominal ultrasonography and a total of 62 twin-bearing ewes were selected. All ewes received a basal pelleted diet (DM at 89%; metabolisable energy: 12.7 MJ/kg, crude protein: 16.9%, fat: 2.2%, crude fibre: 11.2%, salt: 1.5%, acid buffer: 0.8%, lasalocid sodium: 34 mg/kg; Laucke Mills, Daveyston, South Australia, Australia) from ram introduction until parturition. Ewes in the control (CTL, *n* = 20) treatment received the basal diet only whilst treated ewes received betaine from either early in gestation (EB; 2 g/ewe/day throughout gestation, *n* = 21) or late in gestation (LB; 4 g/ewe/day from Day 80 of gestation, *n* = 21). Betaine was included in the basal pellets in the form of betaine 96% (betaine anhydrous, Aocter Group Technology, Liaocheng City, China) at a dose rate of 10 kg/tonne. The selected doses of betaine were based on previous studies in non-pregnant ewes [13] and pregnant sows [18,26]. The administration time of the early betaine treatment was designed to not only increase fetal creatine levels but also to maximise conceptus rate and improve fetal development during early gestation. As is typical for Australia, ewe mating and early pregnancy occurred during summer, and since 4 g/day of dietary betaine has demonstrated adverse effects on the physiological state of sheep during summer [13], this dose was not fed during early pregnancy. The start of the 4 g/day late betaine treatment was designed to occur during late gestation where fetal development predominates [10]. Further, this supplementation period also coincides with the time when sheep producers manage their single- and twin-bearing ewes separately to meet their optimum nutrition profiles [27]. Ewes were fed their pellet ration once daily at approximately 0900 h, along with barley grain, peas and oaten hay to meet nutritional requirements. The quantity of pellets per ewe was calculated based on live-weight. Ewes had *ad libitum* access to clean water throughout the trial. Ewes were housed in their treatment groups and fed via feed trays in paddocks until Day 110 of gestation and were then moved to smaller outdoor pens (8 × 10 m) and housed in groups with an average of seven ewes/pen. At Day 130, ewes were moved indoors and penned individually to allow for easy collection of intensive pre- and post-partum measurements. Once indoors, daily feed residuals were recorded until parturition and treatment diets ceased following parturition. Ewes were weighed and condition scored every two weeks from ram introduction until three days post-partum. General ewe and lamb health were monitored daily until lamb weaning, and if required, animals were treated in accordance with established animal health procedures. At 53.2 ± 0.2 days of age the lambs were de-tailed, castrated (males only) and vaccinated (marking) and weaned at 99.3 ± 0.2 days of age.

### 2.2. Experimental Measures

#### 2.2.1. Parturition Measurements

Starting on Day 147 of gestation, ewes were monitored 24 h/day. To aid in identifying ewes in labour, parturition was video recorded using HiWatch^®^ cameras. Parturition difficulty was scored from 1–3 based on [28], where 1 = no assistance required, 2 = minor assistance given, and 3 = lamb delivered manually. Intervention procedures were only conducted on ewes that indicated distress, or failed to present a lamb within 2 h of the first visible sign of the amniotic sac, or, if there were no signs of the second lamb within 45 min after the firstborn. If required, an internal examination was performed to determine lamb presentation based on definitions adapted from [28]. If the lamb was in the correct position (forelegs first with head resting over knees) the ewe was left unassisted. Only a mal-presented lamb (head only presented with one or both forelimbs retracted, head back, backwards or breech) was corrected and the ewe was left unassisted; however, if the lamb was not born within the following 30 min it was delivered manually.

At the first sign of parturition (i.e., pawing, mucus, fluids, visible sac) a 10 mL colostrum sample was collected, which involved spraying the ewe’s teats with 70% ethanol and removing an equal amount of colostrum from both teats. Gestation length, time of birth, duration and difficulty of parturition, birth order and lamb meconium staining were recorded. Degree of lamb meconium staining was scored from 1–4, where 1 = no staining, 2 = light, 3 = moderate, and 4 = severe. Lambs that failed to suck colostrum within 8 h were bottle-fed and fostered, and removed from the trial.

#### 2.2.2. Post-Partum Measurements

At 4 h post-partum, lamb sex, weight and rectal temperature were recorded and a blood sample was collected by jugular venipuncture using a 21 g needle and 5 mL syringe. Glucose concentration was immediately measured (Abbott Freestyle Optium Neo^®^, Melbourne, Victoria, Australia) using 5–10 µL of whole blood, as well as blood chemistry (lactate, blood urea nitrogen and creatinine) using an EPOC blood analysis system sourced from Siemens Healthineers^®^ (Ottawa, ON, Canada). These blood analysis devices have been validated for use in sheep [29,30,31] and were calibrated in accordance with the manufacturer’s instructions. The blood samples were then immediately dispensed into CAT vacutainer tubes (BD Vacutainer^®^, Oakville, ON, Canada). Samples were stored at 4 °C for 24 h and then centrifuged at 1509 g for 15 min, after which serum was evenly distributed into 1.5 mL tubes and stored at −20 °C until IgG analysis. Lambs were then ear-tagged and marked with a one or two on their back based on birth order for identification purposes. Rectal temperatures were collected at 8, 12, 16 and 20 h post-partum. At 24 h, lamb weight and rectal temperature were recorded and a 5 mL blood sample was collected and processed using the same method detailed above at 4 h. At 72 h, ewe weight and CS, and lamb weight were measured. Each ewe and her lambs were then moved to an outdoor holding pen adjacent to the indoor facility or, if post-nightfall, moved the following morning. At Day 7 post-partum, lamb weight was recorded. Once all lambs had reached seven days of age, ewes and lambs were released into a paddock. Lamb weight was measured at marking and again at weaning.

#### 2.2.3. Lamb Behavioural Analysis

Lamb behavioural latencies (standing attempts, time to stand, sucking attempts and time to suck) were determined by a single operator by reviewing video footage from parturition. These latencies were analysed in accordance with [32] (Table 1).

#### 2.2.4. Immunoglobulin G (IgG) Analysis

Ewe colostrum samples and lamb serum samples were analysed for IgG concentration using a previously validated radial-immunodiffusion (RID) assay developed at the University of Adelaide’s Veterinary Diagnostic Laboratory (Roseworthy Campus, Roseworthy, South Australia, Australia). Agar plates (1% agarose solution in PBS, pH 7.4) containing antibody to ovine IgG (rabbit anti-sheep IgG) were inoculated with serum samples (1:80 in phosphate buffer saline (PBS)), or colostrum samples (1:100 in PBS) and three known purified ovine IgG standard concentrations (0.5, 0.25 and 0.125 mg/mL). Plates were incubated at 24 °C for 48 h for the antigen to bind to the ovine antibody and form a precipitin ring. The diameter (mm) of the precipitin circle was measured using digital fine-scale callipers. Standard curves generated from known concentrations of purified IgG were used to determine IgG concentration of unknown samples. 

#### 2.2.5. Statistical Analysis

Statistical analysis was performed using IBM SPSS Statistics 25 software. Ewe measures (gestation length, duration and difficulty of parturition, colostrum composition) and lamb physiological measures (meconium staining, weight, behavioural latencies (log10 transformed), rectal temperature, serum IgG concentration and blood analysis parameters) were analysed using a General Linear Model (fixed factor: betaine treatment and first-order interactions of betaine treatment with birth order and sex). Repeated measures for lamb weight and rectal temperature were performed using the same General Linear Model for stand-alone variables. A bivariate Pearson Correlation was used to analyse meconium staining, weight, average daily gain, behavioural latencies, colostrum composition and lamb serum IgG. Lamb survival was analysed using Pearson Chi-square test. Unless otherwise stated, results are presented as a mean ± standard error of the mean (SEM) for the main effect of betaine treatment, and those interactions of betaine treatment with other fixed effects that are of interest also given. The number of ewes included for analysis in the CTL, EB and LB treatments were 14, 18 and 19 respectively. The number of lambs included for analysis in the CTL, EB and LB treatments were 28, 36 and 38 respectively. Data from ewes that were culled due to poor health status, aborted or were euthanized prior to parturition (*n* = 9) and triplet-bearing ewes (*n* = 2) and their corresponding lambs (*n* = 6) were excluded from the analysis. Significance was accepted when *p* ≤ 0.05, with *p* < 0.1 considered a tendency. Due to a lack of significance observed for the first-order interactions of betaine treatment with birth order and sex, only the results for the main effects of betaine treatment are presented.

## 3. Results

### 3.1. Gestation Length, Duration and Difficulty of Parturition, Lamb Meconium Staining and Birth Interval

Mean gestation length, duration and difficulty of parturition, lamb meconium staining, and the interval from the start of parturition until birth of the first-born lamb were unaffected by treatment (Table 2). However, the interval between birth of the first- and second-born lamb tended to be longer for EB lambs compared with CTL and LB lambs (*p* = 0.097). No significant interaction was observed for treatment with sex or birth order for lamb meconium staining.

### 3.2. Lamb Weight and Average Daily Gain

Lamb weights were similar between treatments at all time points except at weaning, when EB lambs were heavier than both CTL and LB animals (*p* < 0.05; Table 3). Similarly, when lamb weights were analysed as a repeated measure it was determined that EB lambs were heavier than both CTL and LB animals (*p* < 0.001). Average daily gain (ADG) was similar between treatments from 4 to 72 h post-partum and from 4 h to Day 7 post-partum. However, EB lambs grew faster than CTL and LB lambs from 4 h post-partum to both marking and weaning (*p* = 0.05; Table 3). None of the interactions of treatment with other fixed effects were significant for weight and ADG between all time points.

### 3.3. Lamb Behaviour and Colostrum and Serum IgG

Standing and sucking attempts, and time to suck from standing were unaffected by treatment (Table 4). However, latency to stand from birth was longer in EB compared with CTL and LB lambs (*p* < 0.05; Table 4) and LB lambs were quicker to suck from birth than EB animals (50.88 ± 11.59 vs. 90.20 ± 13.23 min, respectively; *p* < 0.05; Table 4). There was a significant interaction (*p* = 0.031) between treatment and sex for time to suck from standing. CTL females were quicker to suck from standing than EB females and CTL males, and LB females were quicker to suck from standing than EB females, but this did not differ with other treatments with sex (Estimated means ± SEM for female CTL, EB and LB lambs were 17.74 ± 10.21, 45.98 ± 8.87 and 14.76 ± 9.74 min, respectively; Estimated means ± SEM for male CTL, EB and LB lambs were 52.83 ± 13.24, 33.32 ± 12.37 and 26.74 ± 10.38 min, respectively).

IgG concentration (mg/mL) in colostrum collected at parturition and in lamb serum collected at 4 and 24 h were unaffected by treatment (Table 4). No significant interaction was observed for treatment with sex or birth order for lamb serum IgG concentration at both time points.

### 3.4. Plasma Glucose and 4 h Blood Chemistry Analysis

Lamb plasma glucose concentrations (mmol/L) at 4 and 24 h post-partum were unaffected by treatment (Table 5); however, there was a significant interaction (*p* = 0.046) between treatment and birth order at 24 h. First-born EB lambs had higher glucose levels than second-born EB lambs and second-born CTL lambs had higher concentrations than second-born EB and LB lambs, but this did not differ with other treatments with birth order (Estimated means ± SEM for first-born CTL, EB and LB lambs were 5.54 ± 0.50, 6.29 ± 0.41 and 5.88 ± 0.41 mmol/L, respectively; Estimated means ± SEM for second-born CTL, EB and LB lambs were 6.67 ± 0.45, 4.90 ± 0.53 and 5.23 ± 0.46 mmol/L, respectively). There was a tendency for lactate levels to differ between treatment (*p* = 0.063), and blood urea nitrogen (BUN) levels were lower in LB lambs compared to CTL and EB animals at 4 h post-partum (*p* < 0.05; Table 5). Creatinine concentration was unaffected by treatment (Table 5).

### 3.5. Lamb Thermoregulation

Lamb rectal temperatures from 4 to 24 h post-partum were unaffected by treatment (Table 6). However, when lamb rectal temperatures were analysed as a repeated measure, it was determined that CTL lambs were warmer than EB and LB lambs (*p* = 0.012). Further, there was a significant interaction (*p* = 0.028) between treatment and sex at 8 h. CTL females were warmer than CTL males, EB females and males and LB males, but this did not differ with other treatments with sex (Estimated means ± SEM for female CTL, EB and LB lambs were 39.3 ± 0.1, 38.8 ± 0.1 and 39.0 ± 0.1 °C, respectively; Estimated means ± SEM for male CTL, EB and LB lambs were 38.8 ± 0.2, 38.8 ± 0.2 and 38.7 ± 0.1 °C, respectively).

### 3.6. Lamb Survival

Cumulative survival to 4 h post-partum did not differ between treatments. At 24 h post-partum, there was a tendency (*p* = 0.064) for more LB lambs to be alive than either CTL or EB lambs (Table 7), with more LB lambs alive at 72 h and Day 7 compared with CTL and EB lambs (*p* < 0.05; Table 7). Survival rates were unaffected by treatment at both marking and weaning.

## 4. Discussion

This study determined whether maternal supplementation with dietary betaine during pregnancy would improve twin lamb body weight, thermoregulatory capacity, time to stand and suck, colostrum intake and survival from birth to weaning. Supplementing ewes with 4 g/day of betaine during the second half of pregnancy increased twin lamb survival to Day 7 and shortened the time interval from birth to first suck. Whereas, feeding ewes 2 g/day of betaine for the duration of pregnancy increased post-natal growth at weaning but reduced twin lamb survival rates and increased the time to attain behavioural milestones.

Betaine supplementation during the second half of pregnancy improved early post-natal twin lamb survival rates. The current data indicate that cumulative survival for LB lambs was consistently higher to Day 7 post-partum. Betaine can enhance energy production and neuroprotective capacity of neonates by increasing creatine synthesis via the donation of methyl groups in a transmethylation reaction [11], which in turn, reduces incidences of stillbirths and hypoxic birth injury [17]. This is supported by van Wettere et al. [18] and Cai et al. [21] who demonstrated that betaine-supplemented sows had reduced stillbirth frequency and subsequent live-born piglets had increased energy synthesis at birth. It is, therefore, suggested that the increases in cumulative survival in LB lambs may be the result of increased creatine production. However, since this study did not determine circulatory levels of betaine and creatine in the lambs, future research is required to further elucidate this physiological mechanism.

In contrast, lambs born to ewes supplemented with 2 g/day throughout pregnancy experienced significant increases in losses to Day 7 post-partum. As reviewed by Eklund et al. [11], there is minimal evidence to support betaine negatively impacting animal performance and welfare. Thus, the observed negative trend for cumulative survival in EB lambs is likely to be due to other physiological interactions. Interestingly, despite insignificance, the duration and difficulty of parturition were higher in EB ewes compared with CTL and LB ewes. In addition, the birthing interval between the first- and second-born EB lamb tended to be longer than CTL and LB lambs. This suggests that complications in utero and/or immediately following birth may have occurred for EB ewes and their second-born lamb. Prolonged or traumatic parturition often leads to poor cerebral blood flow and oxygen delivery to the fetus, a process known as hypoxic-ischaemic encephalopathy (HIE). HIE is characterized by metabolic acidosis that impairs brain function and causes long-term neurodevelopment morbidities and mortality, particularly for the second-born lamb [33]. Blood lactate levels and degree of meconium staining are traditionally used as biomarkers of fetal distress and hypoxia [34,35,36]. EB lambs did have higher blood lactate levels and meconium staining scores compared with CTL and LB lambs, although the differences were not significant. Further, while the data suggests that the second-born EB lamb was more likely affected by HIE than the first-born EB lamb, no significant differences were observed between birth order for blood lactate levels and meconium staining. Despite this, we cannot dismiss the possibility that EB lambs experienced some symptoms related to HIE, and thus, caused long-term neurodevelopment morbidities that ultimately resulted in the observed mortalities. However, this suggestion is inconsistent with the study by Refshauge et al. [5] who found that since the majority of these losses did not occur around parturition, they are less likely to be caused by labour difficulties and more likely to be attributed to misadventure and exposure. This conclusion is supported by the findings from several lamb autopsies conducted in the current study, which indicated the cause of death was primarily due to hypothermia. Despite this, although the majority of EB lambs died between 24 h and Day 7 post-partum (approximately 17%), the negative impact of HIE on lamb vigour cannot be discounted. While not investigated in lambs, piglets which experience intrapartum hypoxia and asphyxiation typically possess poor neuro-motor and skeletal muscle activity, are less able to maintain thermogenesis and, as a result, typically ingest insufficient colostrum and are less vital [34]. Taken collectively, it is plausible that intrapartum hypoxia was the underlying cause of the increased mortalities observed in EB lambs.

It was anticipated that betaine supplementation would improve lamb birth weight (BW) due to betaine’s osmolytic properties which improve animal growth and feed efficiency. More specifically, betaine increases intestinal cell proliferation and survival by stimulating epithelium in the gut wall to enlarge and thus increase surface area for nutrient absorption [11,37]. However, neither BW nor subsequent weight to marking were improved. Lamb BW is considered a critical determinant of neonatal survival [38], with optimum BW for Merino lambs ranging between 3.5 to 6.0 kg [39,40]. In the current study, lambs were weighed four hours after birth to avoid disrupting standing and sucking behaviours, and thus, the four-hourly weight is assumed to be indicative of BW. While BW was similar between treatment groups, the lightest and heaviest average weight fell within the range identified as being optimal for survival. As such, it is unlikely that BW in this study would be a predisposing factor for lamb survival. Maintaining adequate ewe energy levels is critical due to the strong correlation between lamb BW and maternal nutrition, particularly during late gestation where fetal growth predominates [10,41]. The optimal nutritional management of ewes in this study may have limited the ability of betaine to influence BW, therefore, the potential for betaine to improve BW of lambs born to nutritionally stressed ewes needs to be examined.

Despite this, betaine supplemented to ewes at 2 g/day throughout pregnancy resulted in lambs which were heavier at weaning, with higher ADG noted from 4 h to both marking and weaning. These results are in agreement with Tsiplakou et al. [42] who reported that lambs born to ewes supplemented with 0.6 g/day of rumen-protected betaine during late gestation were 8.9% heavier at weaning and gained 16.7% more weight from birth to weaning. In addition to improved feed efficiency, betaine can also positively influence osmoregulation by increasing water-binding capacity and retention due to improved movement of water across the epithelium via enhanced intraepithelial ion pumps and/or aquaporins [43]. It is, therefore, possible that betaine could have improved nutrient digestibility and utilisation, water retention, or a combination of both in the neonatal gut, thus explaining the observed increases in body weight and ADG at weaning. On the other hand, it is also possible that these results may be related to the reduced survival rates for this treatment. Since more EB lambs died during the first 72 h of life, the ewes would have had to only feed one lamb thereby eliminating competition for teat access and thus, increasing colostrum intake of the surviving lamb. By removing the less viable lamb in the twin litter, it is not surprising that the remaining twin grew at a faster rate and were heavier when compared to a ewe with two viable twins in the CTL and LB groups. Therefore, further research is required to establish the mechanisms whereby maternal betaine supplementation improves post-natal offspring growth and performance.

In addition to improved survival rates, lambs born to ewes supplemented with 4 g/day of dietary betaine were quicker to contact and suck the udder compared with those born to ewes from the EB treatment. Attaining these behavioural milestones are critical for neonatal success, as delays in these homeothermic strategies increase the risk of mortality due to absence or insufficient colostrum intake [9,40]. This apparent improvement in lamb vigour could be attributed to a betaine-induced increase in creatine, which in turn, could have enhanced ATP levels thus protecting the neonatal brain from intrapartum hypoxic damage. As a result, early post-natal locomotor activity was improved as evidenced by the reduced latency from birth to first suck. However, the behavioural data for EB lambs challenges this conclusion as these animals were slower to stand and suck from birth. Despite this, as aforementioned, the data suggest that this result is less likely to be attributed to the betaine treatment itself and more likely to be related to the negative impacts of intrapartum hypoxia. As a result, this ultimately compromised locomotor activity at birth for EB lambs and thus, would explain the observed behavioural retardations.

While not significant, other factors that may have positively influenced the aforementioned improvement in lamb vigour for LB lambs include BW and thermoregulatory capacity. It is well documented that lamb BW and thermoregulatory ability are inextricably linked, as lighter lambs typically elicit lower rectal temperatures leading to poor vigour and survival [8]. To increase heat production following birth, newborn lambs stimulate non-shivering thermogenesis via brown adipose tissue (BAT) [44]. In large newborn lambs, BAT typically constitutes 2.0–4.5% of body weight; however, smaller lambs have disproportionally lower levels of BAT which impairs their ability to generate heat immediately [40]. Although BW was not significantly different between treatments, LB lambs were heavier than CTL and EB lambs. This suggests that LB lambs may have greater BAT for thermoregulation, which is somewhat supported by their rectal temperatures falling within a normal range of 38.3 to 39.9 °C [45,46]. Despite this, there were no significant differences observed in rectal temperature between treatment groups. Taken together, this may have consequently led to improved lamb vigour and thus, could explain why these animals were quicker to suck.

Although betaine supplementation did not significantly improve colostrum IgG and lamb serum IgG concentrations, no adverse effects were noted. Since the observed values for lamb serum IgG concentration at 24 h were higher than the normal levels of around 20 mg/mL [47], it is concluded that lambs received adequate colostrum. However, given that the IgG levels for 0 h colostrum were well above the normal value of 50 mg/mL for ruminants [48] potentially due to carefully managed ewe nutrition; and that the lambs were individually housed with their mothers and had close and constant access to the teats for sucking, this was not surprising. Despite this, there was no significant correlation between colostrum IgG and lamb serum IgG. Interestingly, both betaine treatments resulted in numerically higher (but not significantly) IgG levels in colostrum than CTL ewes. Our results are consistent with the findings of Tsiplakou et al. [42] and Wang et al. [49] who also reported that dietary betaine supplemented to sheep and cattle significantly improved colostrum quality parameters (i.e., milk yield, fat and protein composition) as well as neonatal immunity attributed to increased total protein and globulin concentrations. Therefore, future research is required to further elucidate this biological mechanism in sheep.

While the current data demonstrated that maternal betaine supplementation during late gestation improved various aspects of neonatal twin lamb survival, the impact of indoor housing on these improvements cannot be discounted. In this study, the environmental conditions at lambing were benign due to minimal exposure to extreme weather, predators and infectious agents. Taken together, these factors provided favourable conditions for lamb survival. This is consistent with the findings of Pollard [50] and Hatcher et al. [51] who demonstrated that twin lamb survival rates increased by 13 to 37% within a sheltered environment. However, despite being indoors, significantly fewer LB lambs (0%) died in the first three days post-partum compared with approximately 14 and 20% of CTL and EB lambs, respectively. It could, therefore, be suggested that the beneficial effects of dietary betaine supplemented at 4 g/day may be more profound when ewes lamb in more challenging conditions. As such, future studies examining the effects of maternal betaine supplementation during late gestation, in conjunction with lamb survival parameters, under outdoor extensive conditions are critical. In addition, the experimental design of the current study did not allow an analysis of the effects of varying betaine dose. Future studies including a 2 × 2 factorial design comparing the effect of 2 and 4 g/day of dietary betaine in late, and possibly early, gestation is required to address this limitation. As aforementioned, supplementing 4 g/day of dietary betaine during early pregnancy has demonstrated negative effects on the physiological state of sheep during summer [13], and was the reason why this dose was not fed during early gestation. Therefore, determining the effect of betaine administration at 2 and 4 g/day from early pregnancy, in ewes joined for an autumn lambing period instead of spring, and examining the effects of 2 g/day in late pregnancy, need to be considered in future trials.

## 5. Conclusions

This study provides preliminary evidence that maternal dietary betaine supplementation during late gestation can reduce lamb mortality, which may improve the productivity and welfare of sheep flocks. Betaine supplemented at 4 g/day in the second half of pregnancy increased twin lamb survival to Day 7 and improved lamb viability at birth (shortened time interval from birth to first suck) potentially due to increased creatine production. Whereas, feeding ewes 2 g/day of betaine throughout pregnancy increased post-natal growth at weaning potentially due to enhanced pre-natal intestinal cell growth and survival. Determining lamb betaine and creatine concentration via plasma or serum analysis warrants further exploration. Additionally, future research is critical to determine the true potential of betaine under field conditions in different breeding seasons and climate zones as well as with different breeds. This study has generated useful information towards developing a nutritional strategy to improve twin lamb viability and survival and has contributed to current knowledge gaps surrounding the efficacy of betaine as a maternal supplement.

## Figures and Tables

**Table 1:** Definition of lamb behaviours recorded from birth. Adapted from [32].

Behaviour	Definition
Stand attempt	Lamb on knees, supports part of its weight on at least one foot
Stand	Lamb supports itself on all four feet for at least 5 s
Suck attempt	Lamb in parallel inverse position, head beneath ewe in udder region, prevented from sucking by ewe movement or leaves udder region within 5 s
Successful suck	Lamb has teat in its mouth, in correct position, appears to be sucking for at least 5 s

**Table 2:** Gestation length, duration and difficulty of parturition, birth interval and lamb meconium staining. Data are least-squares mean ± SEM.

	*n*	Treatment			*p*
		Control	Early Betaine	Late Betaine	
Gestation length (days)	51	150.1 ± 0.5	151.1 ± 0.5	149.8 ± 0.5	0.184
Duration of parturition (min)	51	145.2 ± 13.7	175.5 ± 19.1	151.1 ± 12.5	0.339
Difficulty of parturition	51	1.17 ± 0.12	1.26 ± 0.09	1.05 ± 0.10	0.189
Meconium staining	96	1.64 ± 0.22	2.14 ± 0.19	1.61 ± 0.18	0.125
**Birth interval (min)**					
First-born lamb from start of parturition	51	120.1 ± 14.7	107.4 ± 12.8	120.9 ± 13.4	0.722
Between first- and second-born lamb	50	23.2 ± 5.5	36.4 ± 5.4	20.8 ± 5.1	0.097

**Table 3:** Lamb weight at 4 h, 24 h, 72 h, Day 7 post-partum, marking (53.2 ± 0.2 days of age) and weaning (99.3 ± 0.2 days of age); and average daily gain (ADG) from 4 h to 72 h, 4 h to Day 7, 4 h to marking and 4 h to weaning (Estimated marginal means ± SEM). Values with differing superscripts vary significantly (*p* < 0.05).

Lamb Weight (kg) at	*n*	Treatment			*p*
		Control	Early Betaine	Late Betaine	
4 h	98	4.48 ± 0.17	4.51 ± 0.15	4.57 ± 0.13	0.883
24 h	92	4.62 ± 0.18	4.69 ± 0.17	4.72 ± 0.14	0.954
72 h	87	5.19 ± 0.21	5.13 ± 0.20	5.29 ± 0.18	0.747
Day 7	85	6.36 ± 0.24	6.27 ± 0.24	6.25 ± 0.19	0.902
Marking	80	16.71 ± 0.66	18.35 ± 0.64	17.15 ± 0.58	0.106
Weaning	79	26.85 ± 0.94 ^a^	30.35 ± 0.91 ^b^	27.49 ± 0.83 ^a^	0.006
**ADG (kg/day) from**					
4 h-72 h	80	0.24 ± 0.07	0.20 ± 0.07	0.11 ± 0.07	0.393
4 h-Day 7	80	0.26 ± 0.02	0.24 ± 0.02	0.27 ± 0.02	0.820
4 h–marking	80	0.23 ± 0.01 ^a^	0.25 ± 0.01 ^b^	0.23 ± 0.01 ^a^	0.050
4 h–weaning	80	0.22 ± 0.01 ^a^	0.26 ± 0.01 ^b^	0.22 ± 0.01 ^a^	0.016

**Table 4:** Lamb behavioural latencies (log_10_ transformation) recorded shortly after birth (Estimated marginal means ± SEM); and IgG concentration in 0 h colostrum (parturition), 4 h and 24 h lamb serum (Estimated marginal means ± SEM). Values with differing superscripts vary significantly (*p* < 0.05).

Behavioural Latencies	*n*	Treatment			*p*
		Control	Early Betaine	Late Betaine	
Standing attempts (n)	88	12.76 ± 1.38	12.47 ± 1.29	11.38 ± 1.13	0.616
Time to stand (min)	92	32.68 ± 8.10 ^a^	55.26 ± 7.88 ^b^	34.52 ± 6.68 ^a^	0.026
Sucking attempts (n)	83	6.06 ± 0.77	4.84 ± 0.73	4.93 ± 0.68	0.760
Time to suck from birth (min)	91	67.96 ± 13.61 ^ab^	90.20 ± 13.23 ^b^	50.88 ± 11.59 ^a^	0.006
Time to suck from standing (min)	92	35.28 ± 8.36	39.65 ± 7.61	20.75 ± 7.12	0.634
**IgG (mg/mL)**					
0 h colostrum	43	78.03 ± 9.06	93.36 ± 8.11	84.52 ± 7.85	0.449
4 h lamb serum	81	2.40 ± 0.68	1.50 ± 0.62	3.12 ± 0.69	0.208
24 h lamb serum	72	26.69 ± 3.53	27.48 ± 3.69	24.27 ± 3.73	0.753

Definition of lamb behaviours recorded from birth. Adapted from [32]. Stand attempt: Lamb on knees, supports part of its weight on at least one foot; Stand: Lamb supports itself on all four feet for at least 5 s; Suck attempt: Lamb in parallel inverse position, head beneath ewe in udder region, prevented from sucking by ewe movement or leaves udder region within 5 s; Successful suck: Lamb has teat in its mouth, in correct position, appears to be sucking for at least 5 s.

**Table 5:** Lamb plasma glucose and 4 h blood lactate, blood urea nitrogen (BUN) and creatinine (Estimated marginal means ± SEM). Values with differing superscripts vary significantly (*p* < 0.05).

Blood Metabolic Parameters	*n*	Treatment			*p*
		Control	Early Betaine	Late Betaine	
4 h glucose (mmol/L)	97	5.71 ± 0.51	4.53 ± 0.45	5.30 ± 0.41	0.239
4 h blood lactate (mmol/L)	32	3.70 ± 0.48	5.24 ± 0.39	4.05 ± 0.40	0.063
4 h BUN (mg/dL)	31	18.03 ± 1.75 ^a^	21.66 ± 1.49 ^b^	14.75 ± 1.45 ^c^	0.012
4 h creatinine (mg/dL)	31	2.40 ± 0.22	2.15 ± 0.19	2.33 ± 0.19	0.771
24 h glucose (mmol/L)	90	6.09 ± 0.37	5.72 ± 0.35	5.60 ± 0.32	0.538

**Table 6:** Lamb rectal temperatures from 4 to 24 h post-partum (Estimated marginal means ± SEM).

Rectal Temperature (°C) at Time Points	*n*	Treatment			*p*
		Control	Early Betaine	Late Betaine	
4 h	98	38.9 ± 0.2	39.0 ± 0.2	38.8 ± 0.1	0.810
8 h	94	39.1 ± 0.1	38.8 ± 0.1	38.8 ± 0.1	0.111
12 h	96	39.0 ± 0.1	38.8 ± 0.1	38.8 ± 0.1	0.320
16 h	94	39.1 ± 0.1	38.8 ± 0.1	38.9 ± 0.1	0.218
20 h	91	39.0 ± 0.1	38.8 ± 0.1	38.8 ± 0.1	0.578
24 h	91	39.0 ± 0.1	38.9 ± 0.1	39.0 ± 0.1	0.397

**Table 7:** Cumulative survival from birth to weaning. Numbers in parentheses indicate the number of alive lambs within each individual treatment. Values with differing superscripts vary significantly (*p* < 0.05).

Lamb Survival %	Treatment			*Χ* ^2^	*p*
	Control	Early Betaine	Late Betaine		
*n*	28	36	38		
0 h	96.4 (27)	100.0 (36)	100.0 (38)	2.633	0.268
4 h	92.9 (26)	97.1 (35)	100.0 (38)	2.856	0.240
24 h	89.3 (25)	85.7 (31)	100.0 (38)	5.513	0.064
72 h	85.7 (24) ^a^	80.0 (29) ^a^	100.0 (38) ^b^	7.969	0.019
Day 7	82.1 (23) ^a^	68.6 (25) ^a^	100.0 (38) ^b^	13.616	0.001
Marking *	82.1 (23)	68.6 (25)	86.8 (33)	3.896	0.143
Weaning **	82.1 (23)	68.6 (25)	86.8 (33)	3.896	0.143

* 53.2 ± 0.2 days of age; ** 99.3 ± 0.2 days of age.
